# Role of ribophorin II in the response to anticancer drugs in gastric cancer cell lines

**DOI:** 10.3892/ol.2015.2900

**Published:** 2015-01-27

**Authors:** TEIN-MING YUAN, RUEI-YUE LIANG, PIN JU CHUEH, SHOW-MEI CHUANG

**Affiliations:** 1 Institute of Biomedical Sciences, National Chung Hsing University, Taichung 40227, Taiwan, R.O.C.; 2 Department of Surgery, Feng-Yuan Hospital, Ministry of Health and Welfare, Taichung 42055, Taiwan, R.O.C.

**Keywords:** ribophorin II, P-glycoprotein, cytotoxicity, drug resistance

## Abstract

The identification of prognostic markers and establishing their value as therapeutic targets improves therapeutic efficacy against human cancers. Ribophorin II (RPN2) has been demonstrated to be a prognostic marker of human cancer, including breast and pancreatic cancers. The present study aimed to evaluate RPN2 expression in gastric cancer and to examine the possible correlation between RPN2 expression and the response of cells to clinical anticancer drugs, which has received little research attention at present. The gastric cancer AGS, TMC-1, SNU-1, TMK-1, SCM-1, MKN-45 and KATO III cell lines were used as a model to elucidate the role of RPN2 in the response of cells to six common chemotherapeutic agents, comprising oxaliplatin, irinotecan, doxorubicin, docetaxel, cisplatin and 5-fluorouricil. The functional role of RPN2 was assessed by silencing RPN2 using small interfering RNA (siRNA), and the cytotoxicity was determined by an MTS assay and analysis of apoptosis. Molecular events were evaluated by western blotting. All the anticancer drugs were found to exert a concentration-dependent decrease on the cell survival rate of each of the cell lines tested, although the RPN2 levels in the various cell lines were not directly correlated with responsiveness to clinical anticancer drugs, based on the calculated IC_50_ values. siRNA-mediated RPN2 downregulation enhanced cisplatin-induced apoptosis in AGS cells, but did not markedly decrease the cell survival rates of these cells in response to the tested drugs. Furthermore, RPN2 silencing in MKN-45 cells resulted in no additional increase in the cisplatin-induced apoptosis and survival rates. It was also found that RPN2 depletion increased anticancer drug-mediated cytotoxicity in gastric cancer cell lines. However, the predictive value of RPN2 expression in cancer therapy is questionable in gastric cancer models.

## Introduction

The human ribophorin II (RPN2) gene has been localized to chromosome 20ql2-13.1, a region that is frequently deleted in patients with myeloid malignancies (1–4). The gene, which was cloned in 1987 (5), encodes a type I integral membrane protein that is found only in the rough endoplasmic reticulum (ER). Analysis of the structural and topological features of the gene has revealed RPN2 to be a unique integral rough ER membrane glycoprotein that is involved in translocation and the maintenance of the structural uniqueness of the rough ER (5,6). Subsequent biochemical studies have demonstrated that the RPN2 protein is a component of an N-oligosaccharyl transferase complex that conjugates high mannose oligosaccharides to asparagine residues in the N-X-S/T consensus motif of nascent polypeptide chains (7,8).

In addition to its association with myeloid disorders, RPN2 has been demonstrated to be a prognostic marker of human breast (9) and pancreatic cancers (10). RPN2 has also been revealed to contribute to the resistance of tumor cells to chemotherapeutic agents, including docetaxel and taxane, in animal models of breast (11) and ovarian (12) cancers, and in clinical studies of breast (11) and esophageal squamous cell carcinoma (13). In an RNA interference (RNAi)-based screening study, Honma *et al* identified RPN2 as a molecular target for therapy (11). In this animal model of orthotopically implanted, docetaxel-resistant breast tumors, it was revealed that RPN2 silencing effectively facilitated the accumulation of docetaxel in tumor cells, augmented docetaxel-induced apoptotic cell death, and suppressed tumor growth. These studies indicated that RPN2 confers drug resistance by N-glycosylation, which stabilizes the transporter P-glycoprotein (P-gp) in the cellular membrane, and by regulating antiapoptotic genes. This study further demonstrated that the RPN2 expression status in patients with breast cancer was associated with the response to docetaxel, proposing RPN2 as a candidate predictive marker for resistance to docetaxel-based chemotherapy (11,13).

Future studies on genes involved in clinical anticancer drug resistance offer the possibility of identifying early prognostic markers and developing personalized therapeutic targets that can improve the efficacy of therapies against human cancers. There is little current information regarding RPN2 expression in gastric cancer or a possible correlation between its expression and responses to clinical anticancer drugs. Utilizing gastric cancer cell lines as a model, the present study was undertaken to elucidate the role of RPN2 in the response of cells to six common chemotherapeutic agents.

## Materials and methods

### Cell culture

The human gastric AGS, TMC-1, SNU-1, TMK-1, SCM-1, MKN-45 and KATO III carcinoma cell lines were gifted from Dr. Chun-Ying Wu (Division of Gastroenterology, Taichung Veterans General Hospital, Taichung, Taiwan). Cells were cultured in RPMI-1640 medium (Invitrogen, Carlsbad, CA, USA) supplemented with 10% fetal bovine serum (FBS), 2%, w/v sodium bicarbonate, 0.29 mg/ml L-glutamine, 100 units/ml penicillin and 100 μg/ml streptomycin (Invitrogen) in a humidified 5% CO_2_ incubator at 37°C.

### Antibodies

Specific monoclonal antibodies against RPN2 (H300), P-gp (G-1) and β-actin were obtained from Santa Cruz Biotechnology (Dallas, TX, USA). Polyclonal antibodies against poly(ADP-ribose) polymerase (PARP; catalog no. 9542), caspase 3 (catalog no. 9661) and Bcl-2 (catalog no. 2872), and monoclonal antibodies against p21 (catalog no. 12D1) were obtained from Cell Signaling Technology (Beverly, MA, USA). The monoclonal anti-p53 antibody (catalog no. BP53-12) was purchased from Sigma-Aldrich (St. Louis, MO, USA).

### Treatment

Cells (1×10^5^) were seeded in 6 cm culture dishes and incubated overnight at 37°C in medium containing 10% FBS. Cells then subsequently treated with oxaliplatin (20, 40 and 80 μM), irinotecan (10, 20 and 40 μM), doxorubicin (100, 200 and 400 nM), docetaxel (2.5, 5 and 10 nM), cisplatin (2, 4 and 8 μg/ml) and 5 fluorouricil (5-FU; purchased from Sigma-Aldrich) (50, 100 and 200 μM) for 48 h and cell viability was determined by MTS assay.

### MTS assay

Cells (5 × 10^3^) were seeded in 96-well culture plates and incubated overnight at 37°C in medium containing 10% FBS. At the end of treatment, the cell viability was determined using a rapid, tetrazolium-based MTS colorimetric assay (CellTiter 96 cell proliferation assay kit; Promega, Madison, WI, USA) according to the manufacturer’s instructions. All experiments were performed at least in triplicate on three separate occasions. A dose-response curve was plotted, and the concentration of each drug that resulted in a 50% decrease in color development was calculated and classed as the IC_50_ value for each drug. The data are presented as the mean ± standard deviation.

### Apoptosis determination

Apoptosis was measured using an Annexin V-fluorescein isothiocyanate (FITC) apoptosis detection kit (BD Pharmingen, San Jose, CA, USA). The cells cultured in 6-cm dishes were trypsinized and collected by centrifugation. The cell pellet was washed, resuspended in 1X binding buffer and stained with Annexin V-FITC, according to the manufacturer’s instructions. The cells were also stained with propidium iodide (PI) to detect necrosis or late apoptosis. The distribution of viable (FITC/PI double-negative), early apoptotic (FITC-positive), late apoptotic (FITC/PI double-positive) and necrotic (PI-positive/FITC-negative) cells was analyzed using a Beckman Coulter FC500 flow cytometer (Beckman Coulter, Brea, CA, USA). The results are reported as a percentage of the total cells.

### Transfection of small interfering RNA (siRNA)

The RPN2 siRNA duplex was purchased from Dharmacon Research (Lafayette, CO, USA). The gastric cancer cells cultured in glucose-free Opti-MEM were transfected with the siRNA using Lipofectamine RNAiMAX (Invitrogen), according to the manufacturer’s instructions.

### Western blot analysis

The cell extracts were prepared in lysis buffer, which consisted of 20 mM Tris-HCl (pH 7.4), 100 mM NaCl, 5 mM EDTA, 2 mM phenylmethylsulfonyl fluoride, 10 ng/ml leupeptin and 10 μg/ml aprotinin. Volumes of extract containing equal amounts of proteins were separated by sodium dodecyl sulfate-polyacrylamide gel electrophoresis (SDS-PAGE). The proteins were then transferred onto polyvinylidene difluoride (PVDF) membranes (Millipore, Bedford, MA, USA), and the membranes were blocked, washed, and probed with primary antibodies. The antibodies used were monoclonal antibodies against RPN2, P-gp (G 1), β-actin (C4), p21 and p53, and polyclonal antibodies against poly(ADP ribose) polymerase (PARP), caspase 3, Bcl-2. Subsequent to the removal of the primary antibody by washing, the membranes were incubated with horseradish peroxidase conjugated goat anti-mouse or anti-rabbit secondary antibody (Santa Cruz Biotechnology) for 1 h. The blots were washed again, and were developed using enhanced chemiluminescence (ECL) reagents, according to the manufacturer’s instructions (Millipore).

### Reverse transcription-polymerase chain reaction (RT-PCR) analysis

RNA was isolated from the cultured cells using TRIzol reagent (Invitrogen), according to the manufacturer’s instructions. cDNA was synthesized from 2 μg of total RNA by reverse transcription, using the ImProm-II Reverse Transcriptase kit (Promega, Madison, WI, USA) and oligo(d) 12–18 primers. The resulting cDNA was used for the subsequent PCR assays. RPN2 was amplified by using the primers with the following sequences: Forward, 5′-GCCAGGAAGTGGTGTTTGTT-3′ and reverse, 5′-ACAGAGCGAAGAGCAGAAGC-3′, in conjunction with a thermal cycling program consisting of 95°C for 1 min, 55°C for 1 min, and 72°C for 1 min for 30 cycles. β-actin was amplified as an internal control. The β-actin primers were: Forward, 5′-AGAGCTACGAGCTGCCTGAC-3′ and 5′-CACCTTCACCGTTCCAGTTT-3′.

### Statistical analysis

The differences in the data between the groups were analyzed to determine the significance using the Student’s t-test. P<0.05 was considered to indicate a statistically significant difference.

## Results

### RPN2 expression and anticancer drug-induced cytotoxicity

**Figure 1:**
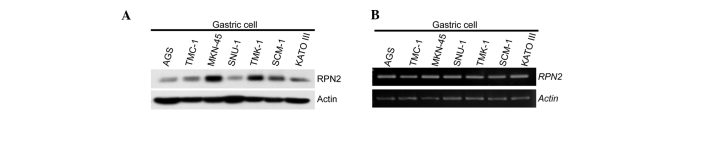
RPN2 expression in the gastric cancer AGS, TMC-1, SNU-1, TMK-1, SCM-1, MKN-45 and KATO III cell lines. (A) The cell extracts were prepared from exponentially growing cells, and extracts containing equal amounts of protein were resolved by SDS-PAGE, followed by western blot analyses using an antibody specific for RPN2. Among these lines, MKN 45 and TMK 1 cells revealed high levels of RPN2 expression at the protein level, whereas AGS and SNU 1 cells exhibited much lower levels of RPN2 protein expression. (B) The RPN2 mRNA levels in each cell line were determined by reverse transcription-polymerase chain reaction using total RNA isolated from cultured cells. RPN2 expression was similar at the transcriptional level in all seven gastric cancer lines.. RPN2, ribophorin II.

It has been proposed that RPN2 expression status is a predictive marker for drug resistance in breast cancer. However, little is known about the correlation between RPN2 expression and the response of gastric cancer cells to clinical anticancer drugs. In the present study, RPN2 expression was analyzed in seven gastric cancer cell lines by western blot analysis (Fig. 1B). Among these lines, MKN-45 and TMK-1 cells revealed high levels of RPN2 expression at the protein level, whereas AGS and SNU-1 cells exhibited much lower levels of RPN2 protein expression (Fig. 1A). Therefore, AGS, MKN-45, TMK-1 and SCM-1 cells were used for the subsequent analysis. Notably, RPN2 expression was similar at the transcriptional level in all seven gastric cancer line (Fig. 1B). The cytotoxicity of six common anticancer drugs, oxaliplatin, irinotecan, doxorubicin, docetaxel, cisplatin and 5-FU, was then determined in these four gastric cancer lines by exposing the cells to various concentrations of anticancer drugs for 48 h and then performing MTS assays, which measured the reduction of the MTS dye to formazan by enzymes in living cells.

**Figure 2:**
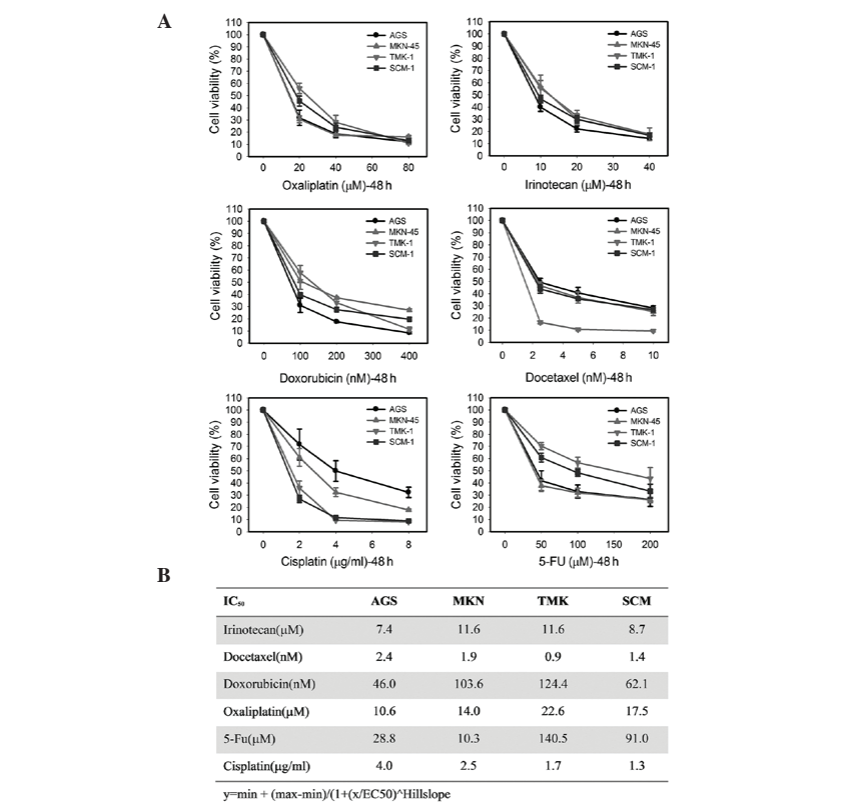
RPN2 expression and anticancer drug-induced cytotoxicity. (A) Cells in the exponential stage of growth were treated with various concentrations 20–80 μM oxaliplatin, 10–40 μM irinotecan, 100–400 nM doxorubicin, 2.5–10 nM docetaxel, 2–8 μg/ml cisplatin and 50–200 μM 5 FU. (B) After 48 h, cell viability was determined by MTS assay and the IC_50_ for each drug in the AGS, MKN-45, TMK-1 and SCM-1 cell lines was calculated. 5-FU, 5-fluorouricil.

In these MTS assays, all six anticancer drugs induced a concentration-dependent inhibition of cell survival in all tested cell lines (Fig. 2A). To evaluate the role of RPN2 in the drug responsiveness of gastric cancer cell lines, the half-maximal inhibitory concentration (IC_50_) was measured for each anticancer drug. The IC_50_ values calculated from the MTS assays, presented in Fig. 2B, indicate a substantial difference in the sensitivity to anticancer drugs of these four cell lines. For example, the AGS cells were moderately resistant to cisplatin exposure compared with the other three cell lines, whereas the SCM-1 cells showed the lowest IC_50_ for cisplatin. By contrast, the AGS cells showed the lowest IC_50_ for irinotecan, doxorubicin and oxaliplatin, whereas the TMK-1 cells exhibited the highest IC_50_ value for these agents out of the four cell lines (Fig. 2B). Compared with the other cell lines, the TMK-1 cells showed the lowest IC_50_ value and were the most sensitive to docetaxel. Additionally, the MKN-45 cells were the most sensitive to 5-FU among the four cell lines with an IC_50_ of 10.3 μM. In contrast, MKN-45 exhibited the most resistanance to irinotecan with an IC_50_ of 11.6 μM. Taken together, the results indicated that RPN2 expression levels were not related to the response to anticancer drugs used in this study.

### Anticancer drug-induced cytotoxicity through apoptosis in gastric cancer cell lines

**Figure 3:**
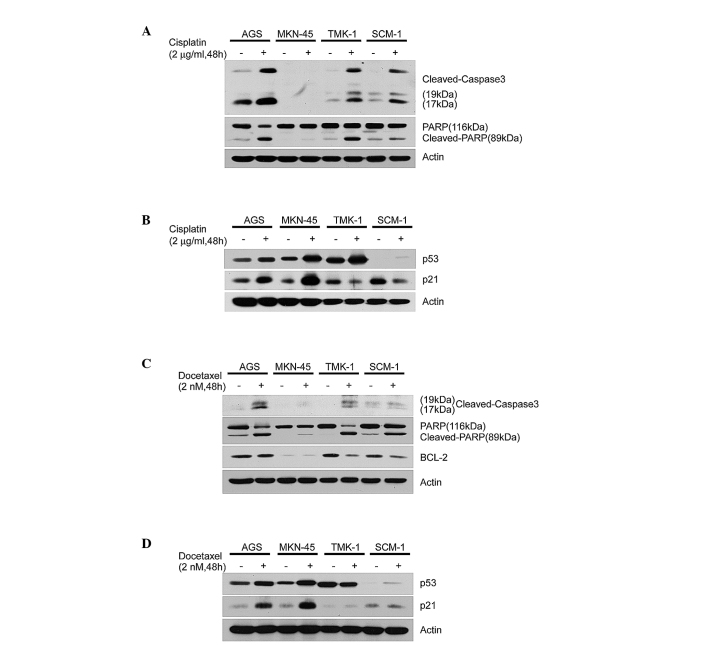
Anticancer drug-induced cytotoxicity determined through assessment of apoptosis in gastric cancer lines. The AGS, MKN-45, TMK-1 and SCM-1 cells were treated with (A and B) 2 μg/ml cisplatin or (C and D) 2 nM docetaxel for 48 h and then the levels of activated caspase 3, cleaved PARP, p53, p21, Bcl-2 and β-actin were detected by western blot analysis. PARP, poly(ADP-ribose) polymerase; Bcl-2, B-cell lymphoma 2.

To further analyze whether the anticancer drug-induced growth inhibition was attributable to apoptosis, the cells were examined for apoptosis-associated protein expression by western blot analysis. At 48 h post-exposure to 2 μg/ml cisplatin, the expression of the cleaved, active form of caspase 3 was significantly enhanced in the AGS, TMK-1 and SCM-1 cells (Fig. 3A). Consistent with this result, 2 μg/ml cisplatin also enhanced PARP cleavage (Fig. 3A). In addition, the expression of p53 protein was induced by 2 μg/ml cisplatin in the AGS and MKN-45 cells, leading to increased p21 expression, indicating a possible p53-mediated growth-inhibition pathway (Fig. 3B). The present study also evaluated the cytotoxic effect of docetaxel and found that docetaxel significantly induced the activation of caspase 3, leading to enhanced PARP cleavage in all cell lines (Fig. 3C). The downregulation of Bcl-2 observed in the TMK-1 and SCM-1 cells is also consistent with the induction of apoptosis caused by 2 nM docetaxel (Fig. 3C). Similarly, 2 nM docetaxel increased the expression of the p53 and p21 proteins in the AGS and MKN-45 cells (Fig. 3D). Notably, although MKN-45 cells exhibited significant induction of p53 and p21 in response to cisplatin and docetaxel, the levels of activated caspase 3 and cleaved PARP were lower compared with other cell lines. In addition, p53 expression was lower in SCM-1 cells compared with the other cell lines, even subsequent to treatment with cisplatin and docetaxel.

### Effect of siRNA-mediated RPN2 silencing on anticancer-drug sensitivity in various gastric cancer cell lines

**Figure 4:**
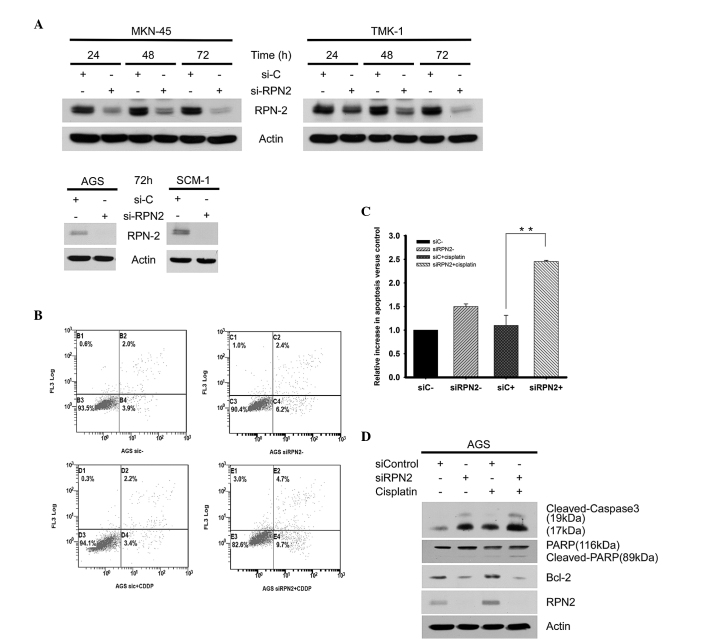
Knockdown of RPN2 enhances apoptotic cell death in the AGS cell line. (A) The MKN-45, TMK-1, AGS and SCM-1 cells were transfected with RPN2 siRNA duplex to specifically silence RPN2 expression. (B) The AGS cells were exposed to 4 μg cispatin for 48 h and the induction of apoptosis was analyzed. The percentage of apoptotic cells was determined by flow cytometry, and the results were expressed as the percentage of total cells in apoptotic populations. (C) Increases in apoptosis were calculated as fold-induction compared to the control, and the values, presented as the mean ± standard deviation, were obtained from at least three independent experiments. (D) The protein levels of activated caspase 3, cleaved PARP, Bcl-2, RPN2 and β-actin in control and RPN2-knockdown cells subsequent to 2μg/ml cisplatin treatment were determined by western blot analysis. ^**^Indicates P<0.01 compared with cisplatin-treated siC. PARP, poly(ADP-ribose) polymerase; Bcl-2, B-cell lymphoma 2; RPN2, ribophorin II; siRNA, small interfering RNA; si-C, control siRNA; si-RPN2, RPN2 siRNA; CDDP, cisplatin.

To directly study the importance of the RPN2 protein level in drug responsiveness of gastric cancer cells, a loss of function approach was employed, using siRNA to knock down RPN2 expression in four gastric cancer cell lines. The RPN2 protein level was markedly downregulated by RPN2 siRNA after 72 h in the tested gastric cancer cell lines (Fig. 4A). The subsequent experiments revealed that siRNA-mediated RPN2 knockdown in the AGS cells increased the percentage of apoptotic cells from 5.9% in the siRNA control cells to 8.6% in the RPN2-knockdown cells. Additional induction of apoptosis was observed after treatment with 4 μg/ml cisplatin, which increased the percentage of apoptotic cells between 5.9% in the control siRNA group and 14.4% in the RPN2-knockdown cells (Fig. 4B). Therefore, knockdown of RPN2 significantly enhanced cisplatin-induced apoptosis in the AGS cells compared with the siRNA control group (Fig. 4C). Furthermore, western blot analyses demonstrated that the depletion of RPN2 increased the level of activated caspase 3 and downregulated Bcl-2 expression, which supports the hypothesis of enhanced induction of apoptosis by RPN2 knockdown alone (Fig. 4D).

**Figure 5:**
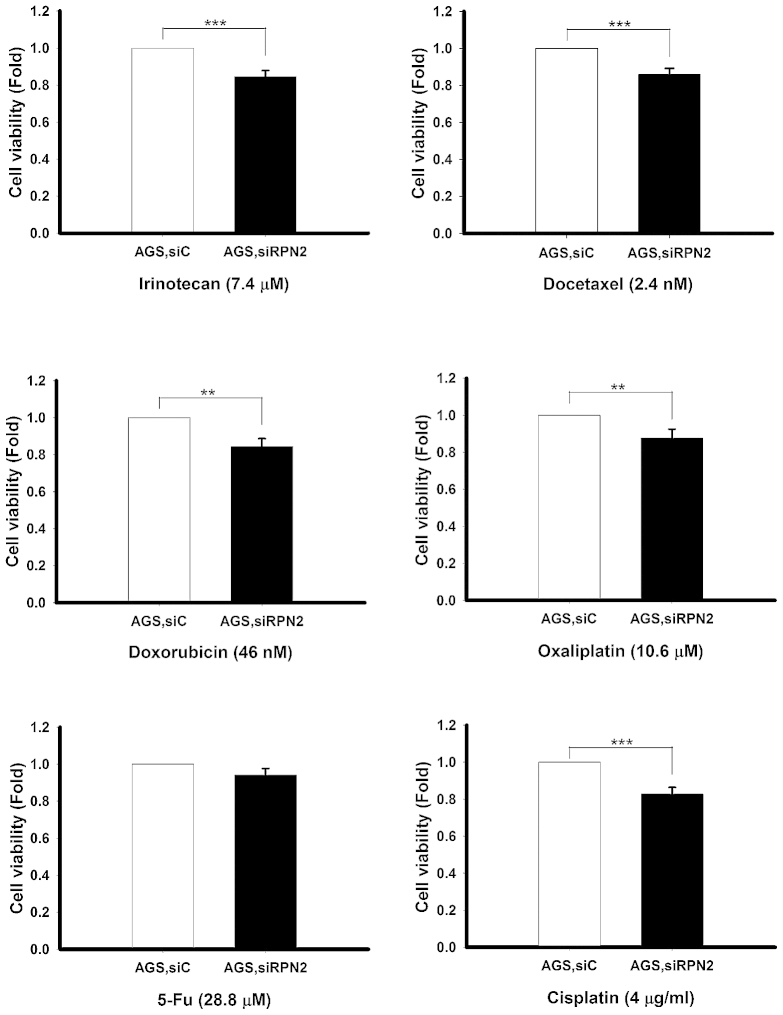
RPN2 silencing enhances anticancer drug-induced cell death in AGS cells. AGS cells were transfected with control siRNA or siRPN2. After 24 h, the IC_50_ of each drug was applied and the cells were incubated for an additional 48 h. MTS assays were performed to determine cell viability. The values were obtained from at least three independent experiments. ^**^Indicates P<0.01 and ^***^ indicates P<0.001 compared with siC. RPN2, ribophorin II; siC, small interfering RNA control; siRPN2, RPN2 small interfering RNA; 5-FU, 5-fluorouracil.

**Figure 6:**
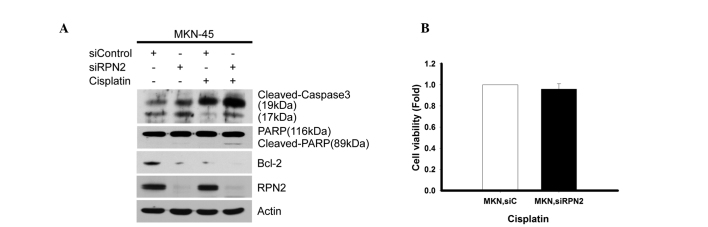
RPN2 silencing does not further increase cisplatin-induced cell death in the MKN-45 cell line. (A) The MKN-45 cells were transfected with siRPN2 to silence RPN2 expression. The protein levels of activated caspase-3, cleaved PARP, Bcl-2, RPN2, and β-actin in control and RPN2-knockdown cells following treatment with 2 μg/ml cisplatin were determined by western blot analysis. (B) The RPN2-knockdown MKN-45 cells were treated with 2.5 μg/ml cisplatin (IC_50_ for MKN-45) for 48 h, and the cell viability was determined using a MTS assay. The values were obtained from at least three independent experiments. PARP, poly(ADP-ribose) polymerase; Bcl-2, B-cell lymphoma 2; RPN2, ribophorin II; si, small interfering RNA; siC, control siRNA; siRPN2, RPN2 siRNA.

The functional significance of RPN2 in cell survival in response to six anticancer drugs was then investigated using MTS assays. The AGS cells were transfected with siRPN2 for 24 h and then treated with anticancer drugs for 48 h. In the presence of anticancer drugs, treatment with RPN2 siRNA slightly reduced the viability of AGS cells relative to the siRNA control. This effect of RPN2-knockdown was significant for all anticancer drugs with the exception of 5-FU, indicating that RPN2 may exert a protective role in cell survival (Fig. 5). RPN2 knockdown in MKN-45 cells also enhanced caspase 3 activation and Bcl-2 downregulation (Fig. 6A). However, subsequent treatment with cisplatin exhibited no evident effect on caspase 3 activation and demonstrated little synergetic effect on Bcl-2 downregulation. These observations were further supported by MTS assays, which revealed no significant decrease in survival in the cisplatin-exposed RPN2-knockdown MKN-45 cells compared with the cisplatin-exposed control siRNA cells (Fig. 6B).

### RPN2-knockdown decreased the level of N-glycosylation on P-gp in response to cisplatin

**Figure 7:**
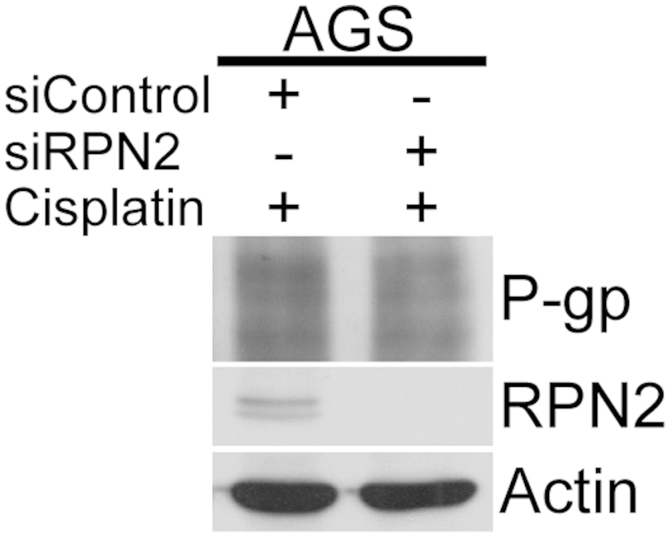
The effect of RPN2 knockdown on the N-glycosylation status of P-gp. RPN2-knockdown AGS cells were treated with 2 μg/ml cisplatin for 48 h. The protein levels of P-gp, RPN2, and β-actin were determined by western blot analysis. P-gp, P-glycoprotein; RPN2, ribophorin II; siControl, control small interfering RNA; siRPN2, RPN2 siRNA.

The potential effect of RPN2 on P-gp 1 function was examined via N-glycosylation in the mechanism of anticancer drug resistance. Expression of the multidrug transporter P-gp, encoded by the multidrug resistance 1 (MDR1) gene, is a major mechanism leading to multidrug resistance in cancer cells. To test the role of P-gp glycosylation in anticancer drug resistance in gastric cancer cells, the AGS cells were transfected with RPN2 siRNA and the glycosylation status of the P-gp protein was determined. A western blot analysis of P-gp revealed that the smear pattern of P-gp, which has previously been demonstrated to reflect the presence of various sizes of intermediately glycosylated forms (11), was slightly decreased upon cisplatin treatment in the RPN2-knockdown cells (Fig. 7).

## Discussion

A previous study revealed that downregulation of RPN2 efficiently induced apoptosis in docetaxel-resistant human breast cancer cells in the presence of docetaxel (11). This study reported that silencing of RPN2 reduced the glycosylation and membrane localization of P-gp, thereby sensitizing cancer cells to docetaxel (11). Considering that numerous anticancer drugs are commonly used in the clinic to treat various human cancers, there is an urgent requirement for an efficient assessment of the curative effects of these agents in individuals. Cell lines are highly useful for preclinical physiological and toxicological studies and are commonly used in a wide range of biomedical studies. Accordingly, the current study used the AGS, SCM-1, TMK-1 and MKN-45 gastric cancer cell lines to investigate whether RPN2 expression is a candidate target for chemotherapy in gastric cancers, one of the most frequent human cancers worldwide. In particular, the role of RPN2 in the efficacy of the clinically used anticancer drugs 5-FU, docetaxel, doxorubicin, irinotecan, cisplatin and oxaliplatin was examined.

Normally, cells possess several mechanisms that protect the cell against a noxious environment. These mechanisms ultimately underlie resistance to cancer chemotherapy. The mechanisms that have been reported to contribute to anticancer drug resistance include decreased drug uptake, increased drug efflux, drug detoxification, induction of anti-apoptotic factors, suppression of pro-apoptotic factors, enhanced DNA repair and increased tolerance to DNA damage (14). Of these, decreases in the intracellular accumulation of hydrophobic chemotherapeutics due to members of the adenosine triphosphate-binding cassette (ABC) transporter superfamily constitute a major mechanism of drug resistance (15). P-gp is one of the key molecules that cause multidrug resistance in cancer cells (16). Overall, the strategy of inhibiting drug efflux transporters, including P-gp, depends on the hypothesis that cancer cells are more dependent on drug efflux or overexpression of the transporter compared with normal cells. In this context, numerous clinical trials of various inhibitors of P-gp have been conducted in an attempt to reverse drug resistance. However, a large majority of these inhibitors have yielded non-significant results, and only a few have demonstrated evidence of a clinical benefit (17,18). Studies of the malignant transformation process have implicated P-gp expression in several oncogene signaling pathways and epigenetic mechanisms (17). It has also been demonstrated that over-activating the P-gp transporter through post-transcriptional modification contributes to increased drug efflux. On the basis of previous studies and the data obtained in the present study, it is indicated that knockdown of RPN2 alone may only result in a limited effect on anticancer drug-induced cell death. This limited efficacy reflects that modulation of P-gp function through N-glycosylation is only one of the numerous mechanisms resulting in drug resistance. Other members of the ABC transporter family and non-ABC mediated drug resistance may also contribute to drug resistance in gastric cancers (18).

Tumor progression is driven by a sequence of randomly occurring mutations and epigenetic alterations of DNA that affect the genes controlling cell proliferation and survival, as well as other traits associated with the malignant cell phenotype. Therefore, tumor cells exhibit heterogeneity that is reflected histologically and genetically. In addition, human cancers express multiple redundant drug-resistance mechanisms. Drug resistance acquired by cancer cells is the leading cause of chemotherapy failure. The identification of promising biomarkers for determining the diagnosis or predicting the responsiveness of tumors to anticancer agents is a compelling and urgent objective, as these biomarkers may improve the assessment of individual treatment requirements and aid in the development of molecular-targeted therapies. Provided that the tumor formation process exhibits features that are specific for distinct organs, future studies may aim to identify specific molecular targets responsible for gastric cancers.

To conclude, the commonly used anticancer drugs examined in the present study effectively decreased cell survival rates in all the tested cell lines in a concentration-dependent manner, although the levels of RPN2 in the various cell lines were not generally correlated with responses to clinical anticancer drugs, calculated as the IC_50_. siRNA-mediated RPN2 downregulation increased the sensitivity of the AGS cells to anticancer drug-induced apoptotic cell death. However, the overall decrease in survival produced by RPN2 silencing was modest and varied between the cell lines. In the AGS cells, siRNA-mediated RPN2 knockdown significantly decreased the survival rate compared with the control siRNA for all the tested drugs, whereas RPN2 silencing did not alter the response to cisplatin in MKN-45 cells. Taken together, these data indicate that RPN2 expression may not be a viable, stand-alone target for gastric cancer therapy.

## Abbreviations

RPN2: ribophorin II
MDR1: multidrug resistance 1
P-gp: P-glycoprotein 1
